# Preparation and Characterization of a Glycyrrhizic Acid-Based Drug Delivery System for Allergen-Specific Immunotherapy

**DOI:** 10.3390/nano12010148

**Published:** 2021-12-31

**Authors:** Ekaterina Pashkina, Veronika Evseenko, Natalya Dumchenko, Maxim Zelikman, Alina Aktanova, Maria Bykova, Mikhail Khvostov, Aleksandr Dushkin, Vladimir Kozlov

**Affiliations:** 1 Research Institute of Fundamental and Clinical Immunology, 14, Yadrintsevskaya, 6300099 Novosibirsk, Russia; aktanova_al@mail.ru (A.A.); vakoz40@yandex.ru (V.K.); 2 Institute of Solid State Chemistry and Mechanochemistry, 18, Kutateladze, 630090 Novosibirsk, Russia; evseenko@solid.nsc.ru (V.E.); ghjnbftyu@gmail.com (M.Z.); dushkin@solid.nsc.ru (A.D.); 3 State Research Center of Virology and Biotechnology VECTOR, Rospotrebnadzor, 630559 Koltsovo, Novosibirsk Oblast, Russia; dumchenko@vector.nsc.ru; 4 Department of Medicine, Novosibirsk State University, 2, Pirogova Street, 630090 Novosibirsk, Russia; maria18021997@mail.ru; 5 Institute of Organic Chemistry SB RAS, 9, Lavrentev Prospect, 630090 Novosibirsk, Russia; mihail.hvostov@gmail.com

**Keywords:** glycyrrhizic acid, house dust mite, allergen-specific immunotherapy, drug delivery

## Abstract

The most effective method of treating allergic diseases, aimed not at relieving symptoms, but at eliminating the cause of the disease, is allergen-specific immunotherapy (AIT). To reduce the risk of side effects and improve the delivery of allergens to the mucosa, various delivery systems, such as liposomes, dendrimers, nanoparticles, etc., can be used. To date, there are data on the creation of delivery systems based on glycyrrhizic acid (GA) and its derivatives, but such a delivery system has not been used for allergen-specific therapy until now. It is also known that GA has an anti-inflammatory effect, shifts the balance towards Th1, and increases the number of Treg cells, meaning that it could potentially enhance the anti-allergic effect of AIT and reduce the risk of unwanted side effects. Thus, the study of the immunomodulatory effect of the supramolecular complexes (micelles) of GA with extracts of allergens holds promise for the development of new drugs for AIT.

## 1. Introduction

According to literary sources, glycyrrhizic acid, the main bioactive glycoside of licorice root, has a wide range of pharmacological properties, including anti-inflammatory action and the promotion of sputum discharge, due to which licorice-based drugs are successfully used to treat diseases such as bronchial asthma and allergic rhinitis [1,2,3]. Additionally, in the last decade, it was found that glycyrrhizic acid and its derivatives have a pronounced antiallergic effect, due to which the use of these substances is becoming relevant in the complex therapy of allergic diseases [4,5,6,7,8,9]. In addition, it is known that glycyrrhizic acid and its derivatives have the ability to form intermolecular complexes and micelles, including drug molecules for targeted delivery [10].

Interest in glycyrrhizic acid and its derivatives as a basis for drug delivery systems can be seen in research around the world [11,12,13,14,15,16,17,18,19]. The above studies have demonstrated the high efficiency of micellar systems of glycyrrhizic acid in solubilization, increasing the bioavailability of poorly soluble drugs, and enhancing their pharmacological action. Drugs with different pharmacological actions have been used as “delivered” molecules; however, the study of glycyrrhizic acid complexes with antiallergic drugs has not yet been carried out.

As is widely known, the most effective method of treating allergic diseases, allowing one to change the response of the immune system to the causative allergen and prevent the further development of allergies, is allergen-specific immunotherapy (AIT). Immunological changes occurring in the body during AIT include the restructuring of the immune response from Th2-type to Th1-type, the formation of allergen-specific “blocking” IgG antibodies, as well as the induction of T-regulatory cells [20,21,22]. It is known that glycyrrhizic acid has an anti-inflammatory effect, shifts the balance towards Th1, and increases the number of Treg [5,6], which means that it has the potential to enhance the anti-allergic effect of AIT and reduce the risk of unwanted side effects.

The use of delivery systems during AIT can increase the efficiency of the procedure. Thus, the subcutaneous administration of liposomes containing house dust mite allergens in patients with bronchial asthma showed a decrease in the severity of symptoms, a decrease in the need for symptomatic therapy, and a decrease in the level of specific IgE [23]. Additionally, a study was carried out in a mouse model of intradermal administration of a nanoemulsion complex with T-cell epitopes of the Japanese cedar allergen; after therapy, a significant decrease in specific IgE was observed [24]. The prospects for intranasal administration of allergens in combination with carriers of lipid origin in the treatment of allergic diseases were also assessed. Thus, in a model of allergic rhinitis in mice sensitized to a cat hair allergen, intranasal administration of the allergen in liposomes based on phosphatidylcholine and cholesterol led to a decrease in the severity of clinical symptoms, a decrease in IL-5 expression, and an increase in the expression of suppressive cytokines IL-10 and TGF-β after provocation by the allergen [25]. Similar results were demonstrated in a mouse model of atopic bronchial asthma sensitized to cockroach allergens [26]. Moreover, there is evidence for intranasal allergens used in combination with nanoemulsions being adjuvants [27]. However, at the same time, the literature does not present data on the use of delivery systems for AIT based on glycyrrhizic acid.

The standard option for subcutaneous administration of the allergen carries risks of adverse events associated with injecting the drug. In this regard, the task of searching for new methods of administration appears to be urgent. The use of dosage forms applied to mucous membranes and, in particular, intranasally (mucosal vaccines) is especially promising. The undoubted advantages of such treatment include the absence of risks associated with the injection of the allergen, as well as the ease of therapy, which increases the patient’s adherence to the recommended treatment regimen. In addition, epithelial cells of the nasal mucosa appear to be involved in the capture and presentation of the allergen [28], which makes intranasal administration of the drug even more promising. It is known that glycyrrhizic acid has an anti-inflammatory effect on the cells of the nasal mucosa, lowering the production of pro-inflammatory cytokines [3,4], which may lead to a decrease in the frequency of local adverse reactions during intranasal AIT with supramolecular complexes of glycyrrhizic acid and an allergen.

Thus, based on the analysis of the current state of research, it seems relevant, promising, and scientifically novel to study the biological properties of supramolecular complexes of glycyrrhizic acid with extracts of allergens, especially with one of the most important allergens—the house dust mite allergen.

## 2. Materials and Methods

### 2.1. Materials

Glycyrrhizic acid (GA) was kindly provided by the researchers from the Institute of Solid State Chemistry and Mechanochemistry by the Group of Mechanochemistry of Organic Compounds (Novosibirsk, Russia). The recombinant 34.5 kDa polypeptide Der p 1 (ProSpec Bio, Rehovot, Israel) was used as a house dust mite peptide. As a material for the study, we used cell line RPMI-2650 (Institute of Cytology RAS, Saint Petersburg, Russia), cell line B16 (kindly provided by Dr. G.V. Seledtsova, Laboratory of Cellular Biotechnologies RIFCI, Novosibirsk, Russia), and peripheral blood mononuclear cells (PBMCs) from healthy donors (n = 10). PBMCs were isolated from heparinized venous blood by centrifugation in a density gradient of ficoll–urografin (1.077 g/cm^3^). The Ethical Committee of RIFCI, Russia, approved the study design and the recruitment of donors. Donors provided written informed consent. The relevant guidelines and regulations were followed when performing the experiments.

### 2.2. Light Scattering Assay

Particle size was measured in solutions by multi-angle light scattering on a Photocor Complex (https://www.photocor.ru/products/photocor-complex (accessed on 29 December 2021), Russia). We studied the associates of molecules of peptide Der p 1, GA in aqueous solutions and a solution obtained by mixing the two previous solutions 1:1. In each solution, measurements were carried out two times: 1–5 h after preparation, and one day after preparation. In all cases, doubly distilled water was the solvent. It was also used as a reference. In the latter case, there were no light scattering signals.

In all the studied samples of GA and peptide solutions and their mixtures, we ob-served particles that were many times larger than individual molecules in size. Evidently, these particles were associates of these molecules. This phenomenon has been well studied for solutions of glycyrrhizic acid [15,16,29], but was so far unknown for noted recombinant polypeptide Der p 1.

### 2.3. Cell Cultures

RPMI-2650 were cultured in MEM containing 50 μg/mL gentamicin, 25 μg/mL thienam, and 10% inactivated FCS serum. B16 and PBMCs cells were cultured in RPMI-1640 containing 0.3% L-glutamine, 50 μg/mL gentamicin, 25 μg/mL thienam, and 10% inactivated FCS serum. The cultures were performed in the presence of the Der p 1 in mixture with GA (1:10) in different concentrations. As a control, we used non-treated PBMCs and PBMCs cultured with GA or Der p 1.

### 2.4. Viability Assay

B16 and RPMI-2560 cells (10^4^ cells/well) and PBMCs (10^5^ cells/well) were cultured in a 96-well plate (Corning, NY, USA). The cells were cultured in the presence of various concentrations of GA, Der p 1, or Der p 1:GA (1:10) for 72 h. DMSO was used as a positive control. After 72 h, 10 μL of WST-1 (Takara Bio, Kusatsu, Japan) stock solution was added into each well containing 100 μL of cell suspension. The absorbance was directly read at 450 nm, and the reference was read at 620 nm.

### 2.5. Transport Assay

RPMI 2650 cells were transferred to Transwell TM microporous filters at approximately 4 × 10^5^/well and incubated for 8 days; then, the culture medium was added from the basolateral side and culture was continued for about 14 to 21 days under the above conditions, with the culture medium being replaced every 2–3 days. The determination of permeability on the culture of RPMI 2650 cells should not be carried out until the integrity and functionality of the monolayer has been established. To determine the suitability of the system, the Transepithelial Electrical Resistance (TEER) is determined. A monolayer of RPMI 2650 cells is considered suitable for determining permeability if the value of TEER reaches a plateau and has a value from 75 Ohm/cm^2^ to 180 Ohm/cm^2^. The measurements of the TEER were carried out before and after the introduction of samples.

The permeability of Der p1 across the RPMI-2650 monolayer was studied. Der p 1 or Der p1:GA (10:10) solutions were added to the apical chamber. After the addition of the solutions, samples were taken in the basal chamber after 15, 30, 45, 60, 90, 120, and 180 min. The medium in equal volume to the sample was added to the basal chamber after sampling. Samples were frozen at −20 °C and stored until use. Der p1 concentration was measured in samples using the ELISA technique following the manufacturer’s instructions (Indoor Biotechnologies, Charlottesville, VA, USA).

### 2.6. T Cell Analysis

PBMCs (10^6^ cells/well) were cultured in a 24-well plate (Costar, London, UK). The cells were cultured in the presence of various concentrations of GA, Der p 1 or Der p 1:GA (1:10), for 120 h. To evaluate the percentage of lymphocyte subsets, PBMCs were stained after cultivation with monoclonal anti-human antibodies (CD25-PE/Cy7, CD3-APC, CD4-PerCP/Cy5.5, and CD127-APC/Cy7), all sourced from BioLegend, (San Diego, CA, USA). Analyses were performed using FACSCanto II (Becton Dickinson, Franklin Lakes, NJ, USA) and FACSDiva software (Becton Dickinson, Franklin Lakes, NJ, USA). Treg population was identified as CD3^+^CD4^+^CD25^+/high^CD127^−/low^ cells.

### 2.7. ELISA Assay

PBMCs (106 cells/well) were cultured in a 24-well plate (Costar, London, UK). The cells were cultured in the presence of various concentrations of GA, Der p 1 or Der p 1:GA (1:10). The production of tumor necrosis factor-α (TNF-α) and interferon-γ (IFN-γ) was assessed after 72 h. After incubation, the cells were pelleted by centrifugation, and the supernatants were collected and stored at −20 °C until required for testing. The concentration of the cytokines was determined using an enzyme-linked immunosorbent assay kit (JSC Vector-Best, Novosibirsk, Russia). The optical densities of colored solutions in the wells were measured using a spectrophotometer (Anthos 2020, Anthos Labtec, Salzburg, Austria) at 450 nm.

### 2.8. Statistical Analysis

ANOVA analyses were performed using GraphPad Prism, with Friedman and Dunn’s multiple comparisons tests. A *p*-value < 0.05 was regarded as the minimum criterion for statistical significance.

## 3. Results and Discussion

**Figure 1:**
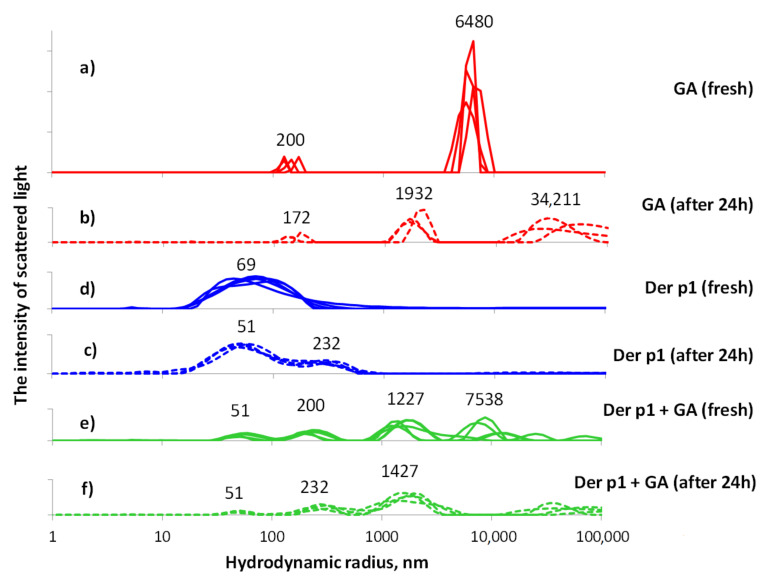
Measurement of particle size in solution by light scattering. We studied aqueous solutions of peptide 10 μg/L (blue curves), GA 100 μg/L (red curves), and a solution obtained by mixing two previous solutions 1:1 (green curves). In each solution, measurements were carried out twice: 1–5 h after preparation (solid line), and one day after preparation (dashed line). All distributions on the graph are normalized. The measurement results show the presence of several sizes of associates in solutions.

We obtained a complex of GA with a peptide of the house dust mite der p 1. According to the data obtained, the delivery system works most efficiently when using a ratio of 10:1 (GA:Der p 1) by mass fraction of the substance (Figure 1). Several measurements were taken for each solution. The resulting distributions are shown in the graph. Peaks from multiple runs do not coincide with each other, and this essentially reflects the heterogeneous sample. After 24 h, the differences between the measurements decrease, which indicates that the equilibrium state of the associates in the solution is approaching. The error of a single measurement is much less than the differences due to the inhomogeneity of the solution. This is due to the measurement technique associated with the accumulation of a sufficient sample (measurement duration is 10 min at a scattered light intensity of about 100,000 count per second), the method for solving the problem of finding the particle size distribution in the polydisperse case, and control of the input data.

We studied aqueous solutions of peptide 10 μg/L (blue curves), GA 100 μg/L (red curves), and a solution obtained by mixing two previous solutions 1:1 (green curves). In each solution, measurements were carried out twice: 1–5 h after preparation (solid line), and one day after preparation (dashed line). All distributions on the graph are normalized. The measurement results show the presence of several sizes of associates in solutions. A day later, the distribution broadens even more, and a second peak appears, which can indicate the deployment of peptide particles in solution. In an aqueous solution of GA, GA molecules form associates 200 ± 70 nm in size, but also much larger associates, larger than 2000 nm, as can be seen in the graph. In an aqueous solution of a peptide with GA, associates of the size of the peptide particles and GA molecule associates are first formed, which indicates their separate presence in the solution. Then, as in a pure GA solution, the peak corresponding to large associates in the graph is replaced by several peaks, which indicates the complication of the branching of the associates. As a result of comparing the peptide solution with GA from their individual solutions, it can be concluded that the processes typical for solutions of individual components prevail in the joint solution. However, this results in associates ranging in size from 700 nm to 3000 nm, which is a wider range than for associates of pure GA. This indicates the incorporation of the peptide into GA associates of a given size as a result of simple mixing of liquid solutions.

**Figure 2:**
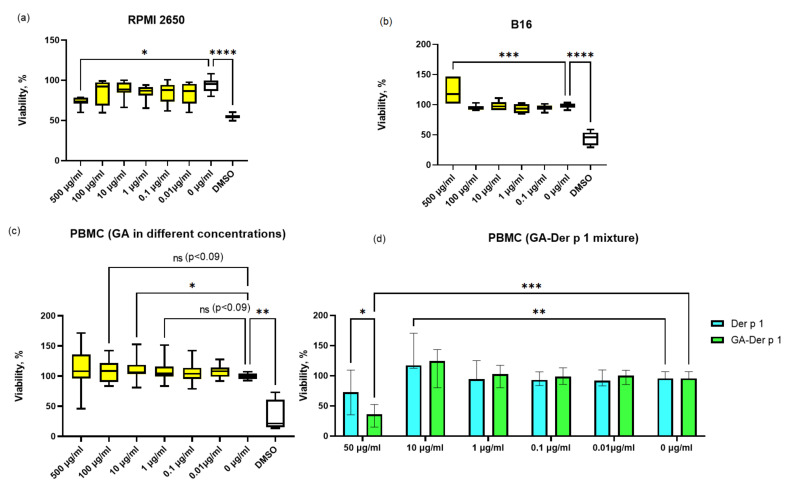
Effects of GA and Der p 1 on cell viability of different cell cultures. (**a**) effects of the GA on cell viability of RPMI-2650 cells. (**b**) effects of the GA on cell viability of B16 cells. (**c**) effects of the GA on cell viability of PBMCs. (**d**) effects of the Der p 1 and Der p 1–GA mixture on cell viability of PBMCs. * Indicates a significant difference (*p* < 0.05); ** indicates a significant difference (*p* < 0.01); *** indicates a significant difference (*p* < 0.001); **** indicates a significant difference (*p* < 0.0001).

First, we investigated in vitro the effect of the glycyrrhizic acid (GA) itself on the viability of various types of cells that can be important during intranasal AIT, namely, cells of the nasal cavity and immunocompetent cells. For this, the effect on the cells of the RPMI 2650 line (nasal carcinoma of the nasal cavity, a standard cell line for creating a model for assessing the penetration of drugs through the nasal mucosa) was evaluated, as well as the primary culture of cells of peripheral blood mononuclear cells (PBMCs) from healthy donors and the B16 mouse melanoma cell line as skin cells. It was found that GA reduces the viability of RPMI 2650 cells at a high concentration of 0.5 μg/mL (Figure 2a). GA did not affect the viability of cells in other concentrations. Additionally, it was shown that GA does not decrease the viability of PBMCs in all concentrations (Figure 2b). Moreover, an increase in the viability of PBMCs was observed upon the addition of GA at a concentration of 10 μg/mL, which is possibly associated with the activation of cells and an increase in their proliferative activity in the presence of GA. In the case of the B16 cell line, GA increased the viability of these cells (Figure 2c), apparently enhancing the proliferation of these cells only at a high concentration (0.5 μg/mL). Consequently, GA has a low effect on the viability of various types of cells.

The next step was a comparative assessment of the effect of the GA complex with the house dust mite allergen Der p 1 (10:1) and the free peptide Der p 1 on the viability of PBMCs (Figure 2d). It was shown that the free peptide and Der p 1–GA mixture increases the viability of PBMCs at a concentration of 10 μg/mL. The increase in viability, apparently indicates the proliferative activity of cells caused by the peptide Der p1. Despite the absence of a history of house dust mite allergy, some seemingly healthy donors can be sensitized to Der p 1, which is able to manifest an increase in proliferative activity. In addition, it was found that at a high concentration, equivalent to 50 μg/mL of the Der p 1 peptide, the complex significantly suppressed the viability of PBMCs, in contrast to the free peptide at the same concentration. Presumably, this effect is associated with an increase in the peptidase activity of Der p 1, which is known as a digestive enzyme of the house dust mite. The mechanisms for enhancing the cytotoxic effect of Der p 1 in the presence of a delivery system such as glycyrrhizic acid are currently not clear and require further study.

**Figure 3:**
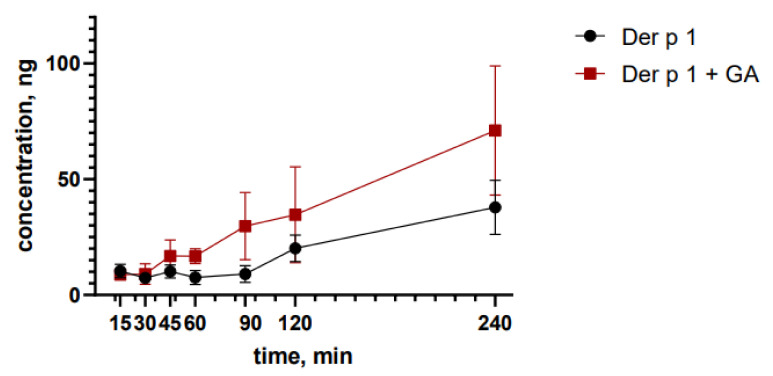
Evaluation of the permeability of Der p 1 across the RPMI-2650 monolayer.

For the next experiments, we selected the highest non-toxic dose of the GA mixture with the Der p 1 peptide—10 μg/mL of the peptide and 100 μg/mL of GA. We studied the effect of GA on peptide Der p 1 transport across the cell monolayer. A significant increase in the transport of the peptide from the complex was found after 120 min and 180 min compared to the free peptide at the same time (Figure 3). It is known that GA can enhance drug transportation across the cell membrane, increasing permeabilization [29]. GA is incorporated into the lipid bilayer of the cell membrane and, thereby, can disrupt its structure and properties [30], therefore affecting the density of intercellular contacts. Therefore, GA can enhance the transport of peptide Der p 1 through the nasal epithelial cells.

**Figure 4:**
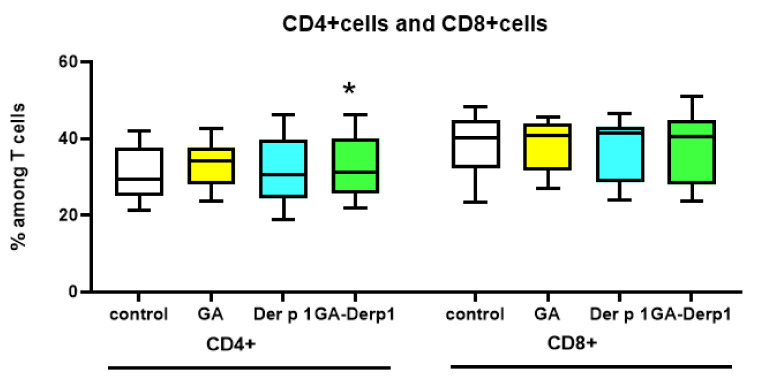
T cell subsets after cultivation with GA–Der p 1 mixture. Data are presented as box-and-whisker plots, with boxes extending from the 25th to the 75th percentile, with a horizontal line at the median, while the whiskers extend to the lowest and highest data points (n = 10). * Indicates a significant difference (*p* < 0.05) vs. control.

The GA–Der p 1 mixture significantly increased the relative number of CD4^+^ cells in comparison to the control in the culture of PBMCs from healthy donors (Figure 4). Adding only GA did not lead to a statistically significant increase in the percentage of CD4^+^ cells; however, there was a tendency (*p* = 0.09) to increase the number of CD4^+^ cells. A similar result may indicate an enhancement of the cellular immune response, since GA is known to direct the immune response towards Th1 [5].

**Figure 5:**
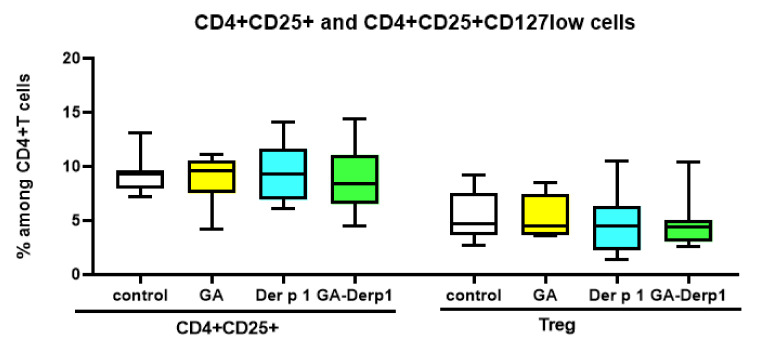
CD4+ cell subsets after cultivation with GA–Der p 1 mixture. Data are presented as box-and-whisker plots, with boxes extending from the 25th to the 75th percentile, with a horizontal line at the median, while the whiskers extend to the lowest and highest data points (n = 10).

**Figure 6:**
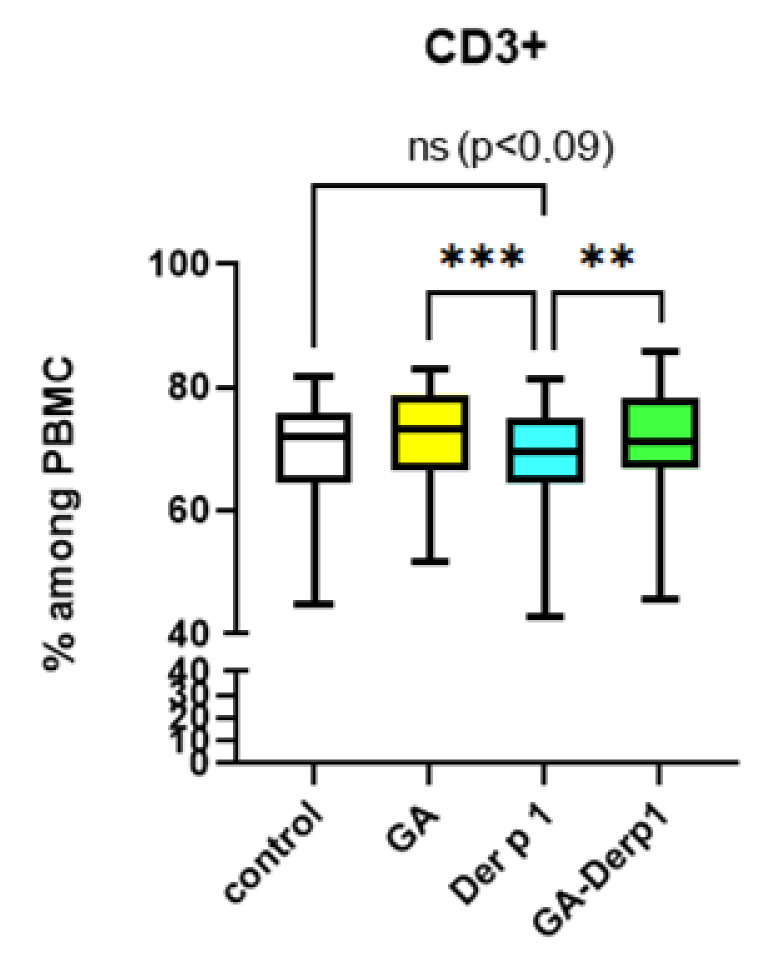
The relative number of CD3+ cells after cultivation with GA–Der p 1 complex. Data are presented as box-and-whisker plots, with boxes extending from the 25th to the 75th percentile, with a horizontal line at the median, while the whiskers extend to the lowest and highest data points (n = 10); ** indicates a significant difference (*p* < 0.01); *** indicates a significant difference (*p* < 0.001).

Neither the complex, nor the free peptide, nor GA influenced the relative number of activated CD4^+^CD25^+^ cells and Treg in the T-helper subpopulation (Figure 5). The free peptide Der p 1 decreased in the relative number of CD3^+^ cells in the PBMC culture (Figure 6). It is possible that the action of the free peptide is associated with Th2 polarization and activation of B lymphocytes, since Der p 1 can shift the balance towards Th2 [31], but this issue requires further study. It is notable that the complex of Der p 1 and GA did not have such an effect, which may indicate the potential of using this complex in AIT due to its influence on the Th1/Th2 balance.

**Figure 7:**
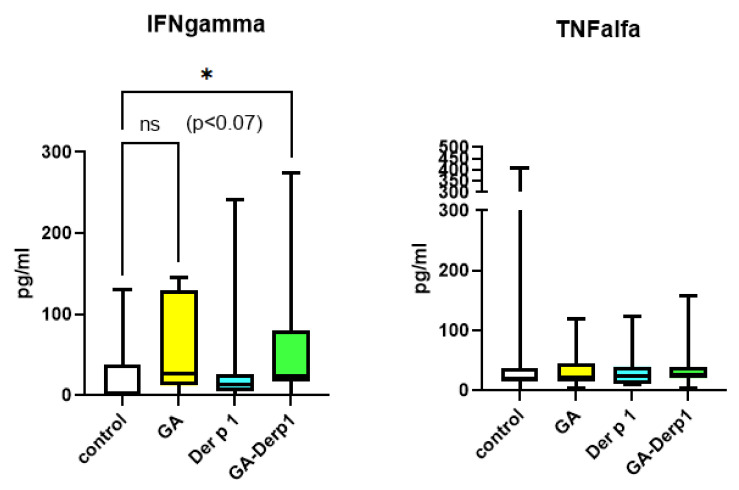
Spontaneous cytokine production by PBMCs cultured in the presence of the GA–Der p 1 complex or its components. Data are presented as box-and-whisker plots, with boxes extending from the 25th to the 75th percentile, with a horizontal line at the median, while the whiskers extend to the lowest and highest data points (n = 7); * Indicates a significant difference (*p* < 0.05).

In vitro studies of spontaneous cytokine production from PBMCs from healthy donors were conducted (Figure 7). Due to the use of primary cultures of healthy donor cells, the results vary greatly, as they are influenced by the characteristics of each individual. Free Der p 1 had no effect on IFN-γ and TNF-α production. The GA and the GA–Der p 1 mixture also had no effect on TNF-α production. The GA–Der p 1 mixture activated the production of IFN-γ, and the concentration of IFN-γ in the presence of free GA also tended to increase compared with that of the control. However, the differences were not significant. The ability to stimulate the production of IFN-γ may indicate the influence on the Th1/Th2 balance and makes the GA–Der p 1 mixture a promising agent for use in allergen-specific immunotherapy.

Fouladi et al. have suggested that GA may have a therapeutic effect on allergic rhinitis, partly by modulation of the Th1/Th2 balance through suppression of OX40 and increasing the activity of regulatory T cells [9]. Tu et al. reported that in the liver and spleen of murine fibrosis models, glycyrrhizin upregulated both Th1/Th2 and Treg/Th17 balances to Th1- and Treg-dominant lineages via multiple pathways such as via c-Jun amino terminal kinase, extracellular signal-regulated kinase and PI3K/AKT [32]. In another study, it was shown that GA reduced airway inflammation and remodeling via the TGF-β1/Smad signaling pathway [33]. The exact mechanism of the immune response modulating in AIT by GA is still unknown and requires further study.

## 4. Conclusions

In the present study, a complex of Der p 1 and GA, obtained here for the first time, demonstrated a low level of toxicity in the studied cell cultures. The results of assessing the effect of the GA complex with Der p 1 on the phenotypic characteristics and cytokine production of PBMCs from healthy donors indicate a change in the Th1/Th2 balance towards the cellular immune response, which can increase the efficiency of the Der p 1 peptide during AIT.

## Data Availability

The data presented in this study is available on request from the corresponding author.
